# Supplementary material for: AI-driven automated discovery tools reveal diverse behavioral competencies of biological networks
Source: eLife. 2025 Jan 13;13:RP92683. doi: 10.7554/eLife.92683 (PMC11729405; doi:10.7554/eLife.92683)
Supplement: Supplementary file 1. [file elife-92683-supp1.docx]

| BioModel ID reference | number of nodes | organism class | systems (observed nodes pairs) |
| --- | --- | --- | --- |
| [BIOMD0000000524](https://www.ebi.ac.uk/biomodels/BIOMD0000000524) | 16 | Homo sapiens | (‘PrER’, ‘mGFP’), (‘DISC’, ‘tBid’), (‘p18inactive’, ‘PrER’), (‘p18inactive’, ‘tBid’), 18inactive’, ‘mGFP’), (‘p18inactive’, ‘PrNES’), (‘DISC’, ‘mCherry’), (‘PrNES’, ‘PrER’), (‘Bid’, ‘mCherry’), (‘tBid’, ‘PrNES’), (‘p18inactive’, ‘PrER_mGFP’), (‘PrNES’, ‘mGFP’), (‘mCherry’, ‘PrER_mGFP’), (‘PrER_mGFP’, ‘PrER’), (‘FADD’, ‘PrNES’), (‘tBid’, ‘mGFP’), (‘DISC’, ‘PrER’), (‘FADD’, ‘mGFP’), (‘mCherry’, ‘mGFP’), (‘FADD’, ‘mCherry’), (‘Bid’, ‘PrER’), (‘FADD’, ‘tBid’), (‘DISC’, ‘p18inactive’), (‘Bid’, ‘PrNES’), (‘p18inactive’, ‘Bid’), (‘tBid’, ‘PrER_mGFP’), (‘FADD’, ‘PrER’), (‘tBid’, ‘mCherry’), (‘PrNES’, ‘PrER_mGFP’), (‘FADD’, ‘p18inactive’), (‘FADD’, ‘Bid’), (‘Bid’, ‘mGFP’), (‘mCherry’, ‘PrER’), (‘PrNES’, ‘mCherry’), (‘DISC’, ‘Bid’), (‘DISC’, ‘PrNES’), (‘Bid’, ‘tBid’), (‘DISC’, ‘PrER_mGFP’), (‘FADD’, ‘DISC’), (‘DISC’, ‘mGFP’), (‘p18inactive’, ‘mCherry’), (‘PrER_mGFP’, ‘mGFP’), (‘FADD’, ‘PrER_mGFP’), (‘tBid’, ‘PrER’) |
| [BIOMD0000000050](https://www.ebi.ac.uk/biomodels/BIOMD0000000050) | 14 | n/a | (‘Gly’, ‘Fru’), (‘FA’, ‘Mel’), (‘FA’, ‘MG’), (‘FA’, ‘Fru’), (‘Mel’, ‘Fru’), (‘Gly’, ‘Mel’), (‘FA’, ‘Glu’), (‘Gly’, ‘AA’), (‘AA’, ‘Fru’), (‘Glu’, ‘Mel’), (‘MG’, ‘Fru’), (‘AA’, ‘Glu’), (‘FA’, ‘AA’), (‘Glu’, ‘Fru’), (‘Gly’, ‘MG’), (‘Glu’, ‘MG’), (‘AA’, ‘MG’), (‘AA’, ‘Mel’), (‘Mel’, ‘MG’), (‘Gly’, ‘FA’) |
| [BIOMD0000000647](https://www.ebi.ac.uk/biomodels/BIOMD0000000647) | 11 | n/a | (‘RKIPP’, ‘MEKPP_ERK’), (‘RKIPP’, ‘RKIPP_RP’), (‘Raf1_RKIP’, ‘ERK’), (‘ERK’, ‘RP’), (‘ERK’, ‘MEKPP’), (‘Raf1’, ‘MEKPP’), (‘MEKPP’, ‘RKIPP_RP’), (‘MEKPP’, ‘RP’), (‘RKIP’, ‘RP’), (‘ERKPP’, ‘RKIPP’), (‘Raf1’, ‘RKIPP’), (‘ERKPP’, ‘RP’), (‘Raf1_RKIP_ERKPP’, ‘ERK’), (‘Raf1_RKIP’, ‘MEKPP’), (‘Raf1’, ‘ERKPP’), (‘ERKPP’, ‘MEKPP_ERK’), (‘ERK’, ‘MEKPP_ERK’), (‘RKIP’, ‘RKIPP_RP’), (‘Raf1’, ‘ERK’), (‘ERKPP’, ‘Raf1_RKIP_ERKPP’), (‘RKIP’, ‘ERKPP’), (‘Raf1_RKIP_ERKPP’, ‘RKIPP_RP’), (‘Raf1_RKIP_ERKPP’, ‘MEKPP’), (‘Raf1_RKIP’, ‘ERKPP’), (‘RP’, ‘RKIPP_RP’), (‘MEKPP_ERK’, ‘RKIPP_RP’), (‘ERK’, ‘RKIPP_RP’), (‘ERKPP’, ‘ERK’), (‘Raf1_RKIP_ERKPP’, ‘RP’), (‘Raf1’, ‘Raf1_RKIP_ERKPP’), (‘Raf1’, ‘Raf1_RKIP’), (‘MEKPP’, ‘MEKPP_ERK’), (‘Raf1_RKIP’, ‘RKIPP’), (‘Raf1_RKIP_ERKPP’, ‘RKIPP’), (‘Raf1_RKIP_ERKPP’, ‘MEKPP_ERK’), (‘MEKPP_ERK’, ‘RP’), (‘RKIP’, ‘MEKPP’), (‘Raf1’, ‘RKIP’), (‘RKIPP’, ‘RP’), (‘RKIP’, ‘MEKPP_ERK’), (‘Raf1_RKIP’, ‘Raf1_RKIP_ERKPP’), (‘Raf1’, ‘RP’), (‘Raf1_RKIP’, ‘RKIPP_RP’), (‘Raf1’, ‘RKIPP_RP’), (‘Raf1_RKIP’, ‘MEKPP_ERK’), (‘ERKPP’, ‘MEKPP’), (‘RKIP’, ‘RKIPP’), (‘Raf1’, ‘MEKPP_ERK’), (‘RKIP’, ‘ERK’), (‘RKIPP’, ‘MEKPP’), (‘ERKPP’, ‘RKIPP_RP’), (‘RKIP’, ‘Raf1_RKIP’), (‘ERK’, ‘RKIPP’), (‘Raf1_RKIP’, ‘RP’), (‘RKIP’, ‘Raf1_RKIP_ERKPP’) |
| [BIOMD0000000520](https://www.ebi.ac.uk/biomodels/BIOMD0000000520) | 3 | Rodents | (‘N0’, ‘N1’), (‘N1’, ‘N2’), (‘N0’, ‘N2’) |
| [BIOMD0000000523](https://www.ebi.ac.uk/biomodels/BIOMD0000000523) | 16 | Homo sapiens | (‘tBid’, ‘mCherry’), (‘PrER’, ‘mGFP’), (‘PrNES’, ‘mGFP’), (‘DISC’, ‘PrNES’), (‘DISC’, ‘tBid’), (‘FADD’, ‘tBid’), (‘p18inactive’, ‘tBid’), (‘FADD’, ‘mCherry’), (‘mCherry’, ‘mGFP’), (‘p18inactive’, ‘mCherry’), (‘tBid’, ‘PrNES’), (‘tBid’, ‘mGFP’), (‘FADD’, ‘DISC’), (‘p18inactive’, ‘mGFP’), (‘mCherry’, ‘PrER’), (‘p18inactive’, ‘PrER’), (‘FADD’, ‘p18inactive’), (‘DISC’, ‘p18inactive’), (‘DISC’, ‘PrER’), (‘FADD’, ‘PrER’), (‘DISC’, ‘mGFP’), (‘PrNES’, ‘mCherry’), (‘p18inactive’, ‘PrNES’), (‘FADD’, ‘PrNES’), (‘tBid’, ‘PrER’), (‘FADD’, ‘mGFP’), (‘DISC’, ‘mCherry’), (‘PrNES’, ‘PrER’) |
| [BIOMD0000000454](https://www.ebi.ac.uk/biomodels/BIOMD0000000454) | 8 | Generic | (‘x3’, ‘y3’), (‘y4’, ‘y2’), (‘y1’, ‘y5’), (‘x3’, ‘y2’), (‘y4’, ‘y5’), (‘x2’, ‘y4’), (‘x1’, ‘y3’), (‘x1’, ‘y5’), (‘y2’, ‘y3’), (‘y1’, ‘y3’), (‘x1’, ‘y4’), (‘x1’, ‘x3’), (‘y4’, ‘y3’), (‘y1’, ‘y2’), (‘y1’, ‘y4’), (‘y1’, ‘x3’), (‘y5’, ‘y2’), (‘y1’, ‘x1’), (‘x2’, ‘x1’), (‘x1’, ‘y2’), (‘x2’, ‘x3’), (‘x3’, ‘y4’), (‘x3’, ‘y5’), (‘y1’, ‘x2’), (‘x2’, ‘y2’), (‘x2’, ‘y5’), (‘x2’, ‘y3’), (‘y5’, ‘y3’) |
| [BIOMD0000000069](https://www.ebi.ac.uk/biomodels/BIOMD0000000069) | 10 | Homo sapiens | (‘srco’, ‘srca’), (‘srci’, ‘srca’), (‘srco’, ‘srcc’), (‘srca’, ‘Cbp_P’), (‘srcc’, ‘Cbp_P_CSK’), (‘srci’, ‘Cbp_P’), (‘srci’, ‘PTP’), (‘Cbp_P’, ‘PTP_pY789’), (‘srcc’, ‘PTP_pY789’), (‘CSK_cytoplasm’, ‘PTP’), (‘srci’, ‘PTP_pY789’), (‘srca’, ‘PTP_pY789’), (‘srcc’, ‘CSK_cytoplasm’), (‘CSK_cytoplasm’, ‘Cbp_P_CSK’), (‘srca’, ‘CSK_cytoplasm’), (‘srca’, ‘PTP’), (‘srcc’, ‘Cbp_P’), (‘srco’, ‘Cbp_P_CSK’), (‘CSK_cytoplasm’, ‘PTP_pY789’), (‘srcc’, ‘PTP’), (‘srca’, ‘Cbp_P_CSK’), (‘srci’, ‘CSK_cytoplasm’), (‘Cbp_P’, ‘Cbp_P_CSK’), (‘Cbp_P’, ‘PTP’), (‘srci’, ‘srco’), (‘srco’, ‘PTP’), (‘CSK_cytoplasm’, ‘Cbp_P’), (‘srca’, ‘srcc’), (‘srci’, ‘Cbp_P_CSK’), (‘Cbp_P_CSK’, ‘PTP_pY789’), (‘PTP’, ‘PTP_pY789’), (‘Cbp_P_CSK’, ‘PTP’), (‘srci’, ‘srcc’), (‘srco’, ‘Cbp_P’), (‘srco’, ‘PTP_pY789’), (‘srco’, ‘CSK_cytoplasm’) |
| [BIOMD0000000455](https://www.ebi.ac.uk/biomodels/BIOMD0000000455) | 9 | Generic | (‘x2’, ‘y5’), (‘y5’, ‘y2’), (‘x2’, ‘y2’), (‘y1’, ‘y3’), (‘x3’, ‘y3’), (‘y1’, ‘x2’), (‘x2’, ‘x1’), (‘x2’, ‘y4’), (‘x3’, ‘y2’), (‘x2’, ‘y3’), (‘y1’, ‘y4’), (‘y5’, ‘y3’), (‘y1’, ‘y5’), (‘x1’, ‘x3’), (‘x1’, ‘y4’), (‘y4’, ‘y5’), (‘x1’, ‘y3’), (‘y1’, ‘x3’), (‘y4’, ‘y3’), (‘x3’, ‘y4’), (‘x3’, ‘y5’), (‘x1’, ‘y5’), (‘y1’, ‘x1’), (‘y1’, ‘y2’), (‘y2’, ‘y3’), (‘y4’, ‘y2’), (‘x2’, ‘x3’), (‘x1’, ‘y2’) |
| [BIOMD0000000526](https://www.ebi.ac.uk/biomodels/BIOMD0000000526) | 16 | Homo sapiens | (‘FADD’, ‘PrER_mGFP’), (‘PrNES’, ‘mCherry’), (‘p18inactive’, ‘PrER_mGFP’), (‘DISC’, ‘p18inactive’), (‘FADD’, ‘p18inactive’), (‘p18inactive’, ‘mCherry’), (‘DISC’, ‘mGFP’), (‘FADD’, ‘mGFP’), (‘p18inactive’, ‘tBid’), (‘p18inactive’, ‘mGFP’), (‘FADD’, ‘DISC’), (‘tBid’, ‘PrER’), (‘DISC’, ‘tBid’), (‘p18inactive’, ‘PrER’), (‘p18inactive’, ‘PrNES’), (‘PrNES’, ‘PrER_mGFP’), (‘PrNES’, ‘PrER’), (‘FADD’, ‘PrNES’), (‘DISC’, ‘PrER’), (‘DISC’, ‘mCherry’), (‘FADD’, ‘tBid’), (‘PrER_mGFP’, ‘PrER’), (‘PrNES’, ‘mGFP’), (‘FADD’, ‘PrER’), (‘mCherry’, ‘mGFP’), (‘DISC’, ‘PrNES’), (‘tBid’, ‘PrNES’), (‘mCherry’, ‘PrER’), (‘PrER_mGFP’, ‘mGFP’), (‘tBid’, ‘mCherry’), (‘FADD’, ‘mCherry’), (‘tBid’, ‘mGFP’), (‘mCherry’, ‘PrER_mGFP’), (‘DISC’, ‘PrER_mGFP’), (‘PrER’, ‘mGFP’), (‘tBid’, ‘PrER_mGFP’) |
| [BIOMD0000000284](https://www.ebi.ac.uk/biomodels/BIOMD0000000284) | 9 | Bacteria | (‘D’, ‘E’), (‘A’, ‘D’), (‘X’, ‘C’), (‘B’, ‘E’), (‘C’, ‘Z’), (‘X’, ‘E’), (‘A’, ‘Z’), (‘X’, ‘Y’), (‘C’, ‘D’), (‘Y’, ‘Z’), (‘E’, ‘F’), (‘F’, ‘Z’), (‘D’, ‘F’), (‘B’, ‘F’), (‘C’, ‘F’), (‘X’, ‘Z’), (‘A’, ‘Y’), (‘X’, ‘D’), (‘A’, ‘F’), (‘B’, ‘Z’), (‘X’, ‘F’), (‘A’, ‘B’), (‘A’, ‘C’), (‘B’, ‘D’), (‘Y’, ‘E’), (‘B’, ‘C’), (‘B’, ‘Y’), (‘C’, ‘Y’), (‘Y’, ‘F’), (‘E’, ‘Z’), (‘X’, ‘B’), (‘D’, ‘Z’), (‘D’, ‘Y’), (‘A’, ‘E’), (‘C’, ‘E’), (‘X’, ‘A’) |
| [BIOMD0000000084](https://www.ebi.ac.uk/biomodels/BIOMD0000000084) | 8 | n/a | (‘Rin’, ‘x2’), (‘x1’, ‘x3’), (‘x2’, ‘x3’), (‘x1’, ‘x2’), (‘Rin’, ‘x3’), (‘Rin’, ‘x1’) |
| [BIOMD0000000052](https://www.ebi.ac.uk/biomodels/BIOMD0000000052) | 11 | n/a | (‘Triose’, ‘lys_R’), (‘Cn’, ‘lys_R’), (‘Acetic_acid’, ‘lys_R’), (‘lys_R’, ‘Melanoidin’), (‘Triose’, ‘Melanoidin’), (‘Cn’, ‘Melanoidin’), (‘Formic_acid’, ‘lys_R’), (‘C5’, ‘lys_R’), (‘C5’, ‘Formic_acid’), (‘Triose’, ‘Acetic_acid’), (‘C5’, ‘Acetic_acid’), (‘Acetic_acid’, ‘Melanoidin’), (‘C5’, ‘Cn’), (‘C5’, ‘Melanoidin’), (‘Cn’, ‘Acetic_acid’), (‘C5’, ‘Triose’), (‘Formic_acid’, ‘Triose’), (‘Formic_acid’, ‘Cn’), (‘Triose’, ‘Cn’), (‘Formic_acid’, ‘Melanoidin’), (‘Formic_acid’, ‘Acetic_acid’) |
| [BIOMD0000000271](https://www.ebi.ac.uk/biomodels/BIOMD0000000271) | 6 | Rodents | (‘EpoR’, ‘dEpoe’), (‘Epo_EpoRi’, ‘dEpoe’), (‘Epo_EpoR’, ‘dEpoe’), (‘Epo_EpoRi’, ‘dEpoi’), (‘EpoR’, ‘dEpoi’), (‘Epo’, ‘dEpoe’), (‘Epo’, ‘dEpoi’), (‘Epo_EpoR’, ‘dEpoi’), (‘dEpoi’, ‘dEpoe’) |
| [BIOMD0000000461](https://www.ebi.ac.uk/biomodels/BIOMD0000000461) | 4 | Bacteria | (‘lacz’, ‘x’), (‘IPTG’, ‘sigb’), (‘sigb’, ‘x’), (‘sigb’, ‘lacz’), (‘IPTG’, ‘x’), (‘IPTG’, ‘lacz’) |
| [BIOMD0000000525](https://www.ebi.ac.uk/biomodels/BIOMD0000000525) | 16 | Homo sapiens | (‘p18inactive’, ‘PrNES’), (‘p18inactive’, ‘PrER’), (‘tBid’, ‘PrNES’), (‘DISC’, ‘PrER’), (‘mCherry’, ‘PrER’), (‘PrNES’, ‘mCherry’), (‘DISC’, ‘tBid’), (‘FADD’, ‘mCherry’), (‘p18inactive’, ‘tBid’), (‘FADD’, ‘PrER’), (‘FADD’, ‘p18inactive’), (‘tBid’, ‘mGFP’), (‘DISC’, ‘p18inactive’), (‘mCherry’, ‘mGFP’), (‘DISC’, ‘PrNES’), (‘PrER’, ‘mGFP’), (‘DISC’, ‘mGFP’), (‘FADD’, ‘DISC’), (‘tBid’, ‘PrER’), (‘p18inactive’, ‘mCherry’), (‘tBid’, ‘mCherry’), (‘PrNES’, ‘mGFP’), (‘PrNES’, ‘PrER’), (‘FADD’, ‘PrNES’), (‘DISC’, ‘mCherry’), (‘p18inactive’, ‘mGFP’), (‘FADD’, ‘tBid’), (‘FADD’, ‘mGFP’) |
| [BIOMD0000000521](https://www.ebi.ac.uk/biomodels/BIOMD0000000521) | 4 | Homo sapiens | (‘P’, ‘Q’) |
| [BIOMD0000000010](https://www.ebi.ac.uk/biomodels/BIOMD0000000010) | 8 | Amphibians | (‘MKK_P’, ‘MAPK’), (‘MKK’, ‘MAPK_PP’), (‘MKK’, ‘MKK_PP’), (‘MKK_P’, ‘MKK_PP’), (‘MKK’, ‘MAPK’), (‘MKK_PP’, ‘MAPK_P’), (‘MAPK’, ‘MAPK_PP’), (‘MKK_PP’, ‘MAPK’), (‘MKK’, ‘MAPK_P’), (‘MKK_PP’, ‘MAPK_PP’), (‘MAPK_P’, ‘MAPK_PP’), (‘MKK_P’, ‘MAPK_PP’), (‘MKK’, ‘MKK_P’), (‘MKK_P’, ‘MAPK_P’) |
| [BIOMD0000000029](https://www.ebi.ac.uk/biomodels/BIOMD0000000029) | 4 | Amphibians | (‘M’, ‘MpT’), (‘M’, ‘MpY’), (‘MpY’, ‘MpT’) |
| [BIOMD0000000197](https://www.ebi.ac.uk/biomodels/BIOMD0000000197) | 5 | n/a | (‘x1’, ‘x2’), (‘x1’, ‘x4’), (‘x1’, ‘x3’), (‘x1’, ‘x5’), (‘x5’, ‘x4’), (‘x3’, ‘x5’), (‘x5’, ‘x2’) |
| [BIOMD0000000272](https://www.ebi.ac.uk/biomodels/BIOMD0000000272) | 6 | Rodents | (‘SAv_EpoR’, ‘SAv_EpoRi’), (‘EpoR’, ‘SAv_EpoR’), (‘EpoR’, ‘SAv_EpoRi’) |
| [BIOMD0000000167](https://www.ebi.ac.uk/biomodels/BIOMD0000000167) | 7 | Generic | (‘Pstat_nuc’, ‘stat_nuc’), (‘stat_nuc’, ‘species_test’), (‘Pstat_nuc’, ‘species_test’) |
| [BIOMD0000000262](https://www.ebi.ac.uk/biomodels/BIOMD0000000262) | 11 | Rodents | (‘Akt’, ‘S6’) |
| [BIOMD0000000240](https://www.ebi.ac.uk/biomodels/BIOMD0000000240) | 6 | Bacteria | (‘DegU’, ‘mDegU’), (‘Dim’, ‘mDegU’), (‘DegUP’, ‘mDegU’) |
| [BIOMD0000000037](https://www.ebi.ac.uk/biomodels/BIOMD0000000037) | 12 | Slime Mold | (‘Xi’, ‘Ya’), (‘Xi’, ‘Pi’), (‘Ya’, ‘Pi’) |
| [BIOMD0000000263](https://www.ebi.ac.uk/biomodels/BIOMD0000000263) | 11 | Rodents | (‘Akt’, ‘pro_TrkA’), (‘S6’, ‘pro_TrkA’), (‘Akt’, ‘S6’) |
| [BIOMD0000000641](https://www.ebi.ac.uk/biomodels/BIOMD0000000641) | 5 | Homo sapiens | (‘CellCact’, ‘Effectoract’) |
| [BIOMD0000000413](https://www.ebi.ac.uk/biomodels/BIOMD0000000413) | 5 | Plants | (‘TIR1’, ‘auxinTIR1’), (‘auxinTIR1’, ‘VENUS’), (‘TIR1’, ‘VENUS’) |
| [BIOMD0000000624](https://www.ebi.ac.uk/biomodels/BIOMD0000000624) | 7 | Homo sapiens | (‘APAP’, ‘APAPconj_Glu’) |
| [BIOMD0000000945](https://www.ebi.ac.uk/biomodels/BIOMD0000000945) | 5 | Homo sapiens | (‘L_m’, ‘H_m’) |
| [BIOMD0000000459](https://www.ebi.ac.uk/biomodels/BIOMD0000000459) | 4 | Bacteria | (‘IPTG’, ‘sigb’) |

Table 1: List of biological networks from Biomodels used in this study. The resulting database includes 30 biological networks (one row per network) and a total of 432 systems, which is defined as a (GRN model, behavior space (Z)) tuple and where the pairs of observed nodes (used as behavior spaces) per network are given in the last column. Please note that we let the following chemical species evolve in time, despite being labeled as "constant" or "boundary" species in the original SBML models: model #262: ['EGF', 'pro_EGFR'], model #284: ['X', 'Y', 'Z'], model #37: ['Gluc'], model #263: ['NGF', 'pro_TrkA'], model #454: ['y1', 'y2', 'y3', 'y4', 'y5'], model #455: ['y1', 'y2', 'y3', 'y4', 'y5', 'y6'], model #459: ['IPTG'], model #461: ['IPTG'], model #624: ['X1'].
